# Microfibrillated cellulose films containing chitosan and tannic acid for wound healing applications

**DOI:** 10.1007/s10856-021-06536-4

**Published:** 2021-06-12

**Authors:** Meysam Aliabadi, Bor Shin Chee, Mailson Matos, Yvonne J. Cortese, Michael J. D. Nugent, Tielidy A. M. de Lima, Washington L. E. Magalhães, Gabriel Goetten de Lima, Mohammadreza Dehghani Firouzabadi

**Affiliations:** 1 Department of Paper sciences and engineering, Gorgan University of Agricultural Sciences and Natural Resources, Gorgan, Iran; 2 Materials Research Institute, Athlone Institute of Technology, Athlone, Ireland; 3 Programa de Pós-Graduação em Engenharia e Ciência dos Materiais - PIPE, Universidade Federal do Paraná, Curitiba, Paraná, Brazil; 4 Embrapa Florestas, Colombo, Brazil

## Abstract

The effectiveness of tannic acid as antimicrobial and wound healing for burns have been shown for a century; however, uncontrolled target dosage may result in undesirable side-effects. Remarkably, tannic acid polyphenols compounds crosslinked with polymeric materials produce a strong composite containing the beneficial properties of this tannin. However, investigation of the crosslink structure and its antibacterial and regenerative properties are still unknown when using nanocellulose by mechanical defibrillation; additionally, due to the potential crosslink structure with chitosan, its structure can be complex. Therefore, this work uses bleach kraft nanocellulose in order to investigate the effect on the physical and regenerative properties when incorporated with chitosan and tannic acid. This film results in increased rigidity with a lamellar structure when incorporated with tannic acid due to its strong hydrogen bonding. The release of tannic acid varied depending on the structure it was synthesised with, whereas with chitosan it presented good release model compared to pure cellulose. In addition, exhibiting similar thermal stability as pure cellulose films with antibacterial properties tested against *S. aureus* and *E. coli* with good metabolic cellular viability while also inhibiting NF-κB activity, a characteristic of tannic acid.

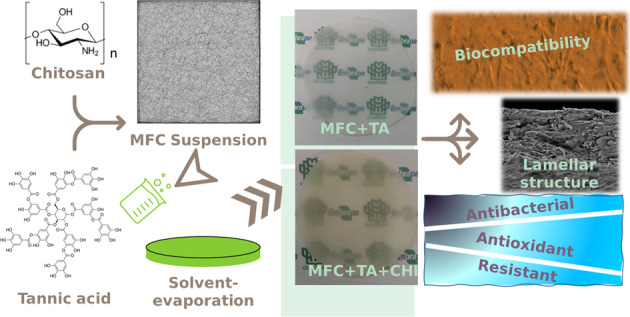

## Introduction

Tannins are phenolic compounds produced in many plants, and they are currently being studied because of their high molecular weight and phenolic hydroxyl groups that are able to crosslink with macromolecules by forming hydrogen bonds with hydrogen-bonding donating polymer [1]. In particular, tannic acid is a promising tannin as it is a water-soluble tannin used as a food additive that exhibits antioxidant and antibacterial properties [2]. Amongst polyphenols, tannic acid is considered to have one of the highest amounts of antioxidant capacity [3]. In addition, because of their ability to crosslink, tannins can protect epithelial tissues due to their antimutagenic, anticarcinogenic, anti-inflammatory and antiviral applications [4].

Treatment using tannic acid has been reported since 1920s for burn injuries, which led to a significant reduction in mortality rates [5]. Tannic acid is known to be antimicrobial to a whole spectrum of microorganisms, and the natural crosslinking ability has been reported for chitosan and cellulose [6, 7]. A combination of tannic acid with nanocrystalline cellulose was also reported previously for chitosan films, which altered their mechanical properties when heat-treated [8].

Many materials used for wound healing are synthesised using nanocellulose due to its high mechanical strength and non-toxic, which can be produced from bacterial cellulose [9] or by nanocrystals formulation [10]. However, recent work from our group has shown that nanocellulose from kraft paper processed by mechanical defibrillation can be a promising alternative for wound healing. This is owing to its good adhesion and moisture retention [11], with a similar healing rate compared to bacterial cellulose films [12]; though actually presenting a better interaction within the wound. The production of this material is very cheap and simple, and the mixture of components that can be done before or after milling demonstrates very different responses; whereas the interaction can be improved when mixing components within the mechanical defibrillation, as shown before for tannins [13].

Chitosan is yet another natural material with antibacterial effect and good regenerating ability [14], even reported to be able to repair the nervous system [15] and effective as a wound healing material [16]. It is possible to modify the functionality of this material due to the amine groups that are highly reactive, and are able to incorporate active compounds or obtaining nanoparticles [17]. Therefore, many works have reported its usage when incorporated with other materials as films [18]. They are also reported in many alternative applications, such as pervaporation dehydration of ethanol and isopropanol [19]. Although chitosan films are very brittle on their own, the addition of tannic acid offers a possibility to improve it by acting as plasticizer [6].

Although the crosslinking ability with both cellulose and chitosan has been reported, the usage of microfibrillated cellulose obtained from kraft paper as solvent cast films are still unknown. Besides, their effect was not yet evaluated under antibacterial and in vitro cell viability when combined to chitosan and tannic acid. Therefore, in this work we evaluate the effect of tannic acid as a crosslinking agent and its antibacterial and cellular regeneration in vitro for wound healing applications for microfibrillated cellulose films and combined with chitosan, which were also investigated when glycerine is added as a plasticizer.

## Materials and methods

### Materials

Materials used in this work were Imortalised human keratinocytes (HaCaT) cell line (Caltag MedSystems Ltd, UK); bleached eucalyptus kraft pulp (Suzano Papel e Celulose, Brazil) for MFC preparation. Tannic acid (Sigma Brazil–ACS reagent product number 403040), Chitosan of low molecular weight containing 50,000–190,000 Da (Sigma Brazil, product number 448869), Glycerine (Dinâmica química contemporânea Ltda., Brazil), *E. coli* from ATCC 25922 and *S. aureus* NCTC 12981.

### Film formation

#### Chitosan solution

To dissolve chitosan, we followed the method described by [6] with slight modifications, chitosan solution of 1% (w/w) was prepared using 1.5% (v/v) of acetic acid under stirring for 24 h.

#### Microfibrillated cellulose gel

The procedure follows the one described before [20]. First, bleached kraft pulp was adjusted to 3 wt%/v (3 g/100 ml) using either distilled water, or the previously chitosan solution of 1% in acetic acid as the solution, and was further fragmented using a 450 W blender for 10 min. Afterwards, it was subjected to further grinding using Super Masscolloider (Masuko Sangyo Co. Ltd., Japan). The technical parameters related to the microfluidizer in obtaining the cellulose nanofibrils were: rotation 1500 rpm; the number of steps 30; distance between discs 0.1 mm. The maximum number of steps was defined as when the cellulose gel, passing through the grinder, would surpass the maximum amperage of the electric system. Therefore, having two sets of gels – a pure MFC using distilled water as the solvent, and another set using the solution of 1% chitosan that contained acetic acid as a solvent. Besides, for plasticized films, glycerine was added as 37 wt%/wt of cellulose (1.11 g glycerol/100 ml of gel solution).

#### Tannic acid

Tannic acid (10% wt. of cellulose) was dissolved within cellulose suspension having a final concentration of 10% (%wt of cellulose) within both sets of gels.

#### Film formation using casting evaporation method

For film formation, 40 ml of each gel was poured on petri dish, which were immediately dried at 60 °C using an Q319V Vacuum Drying Oven (Quimis, Brazil). The samples used in this work follows the denomination of the material used; therefore, MFC for microfibrillated cellulose, CHI for chitosan, TA for tannic acid and GLY for glycerine.

### Scanning electron microscopy (SEM)

The hydrogel morphology was performed using scanning electron microscopy (TESCAN Performance in Nanospace) with the back-scattered electron (BSE) mode. Prior to imaging, the samples were sliced to obtain cross section regions and were also gold-sputtered for coating using Baltec SCD 005 for 110 s at 0.1 mBar vacuum. Images were recorded at an accelerating voltage 20 kV. Coupled with the SEM, Energy Dispersive Spectroscopy was used to analyse one specific sample for confirmation of contamination from Oxford Instruments.

### Fourier transform infra-red spectroscopy (FTIR)

The FTIR spectra were obtained on a Perkin Elmer spectrometer, model FTIR/NIR Frontier, using an attenuated total reflectance (ATR) accessory with zinc selenide (ZnSe) crystal surface. A resolution of 4 cm^−1^ and the arithmetic average of four scans was used in the wavenumber range of 4000–550 cm^−1^. Prior measurements, samples were dried in a vacuum oven at 40 °C until no variation in its weight were detected.

### Thermo and mechanical analysis

For DSC, sample weights between 9–12 mg were measured and encapsulated in alumina sample pans. A temperature ramp from 0 to 400 °C at a rate of 10 °C/min was used with an empty closed alumina pan as a reference. TGA curves were obtained with a heating rate of 10 °C min^−1^ until 600 °C using platinum pans with samples weighing around 3.0 mg. The experiments were carried out under a nitrogen flow of 50 mL min^−1^, in a Q600 TA Instruments.

DMA analyses were performed on DMA Q800 TA Instruments equipment using the film tension clamp. Stress–strain tests were performed with a ramp force of 1 N/min up to 18 N, and the elastic modulus was calculated from the initial linear region – 0.002% strain of the stress–strain curve. All tests from DMA were performed using three scans per sample.

### Drug release

100 mg of the film was immersed for release in 100 mL of DI water at 25 °C. Absorbances at 275 nm were collected at 0, 2.4, 24, 48 and 168 h. The concentration of the solutions was determined on the basis of a tannic acid standard curve. The percentage released was determined based on the concentration of tannic acid present in each film.

### Antioxidant activities (DPPH and ABTS)

All antioxidant activities assays were performed in a spectrophotometer UV/VIS (Shimadzu, model 1800, Kioto, JPN). The antioxidant capacity of extracts by free radical DPPH (2,2-diphenyl-1-picrylhydrazyl) was determined according to the procedure of Siripatrawan and Harte [21] with minor modifications. Firstly, 25 mg of each film sample was extracted in 3 ml of distilled water, for use as a film extract solution. Then, 0.1 mL of the extract solution was added to 3.9 mL of DPPH methanolic solution (60 μmol.L^−1^), and kept under dark for 30 min (reaction) and then the absorbance was measured at 515 nm. The results were expressed as a percentage of radical scavenging.

The free radical scavenging by ABTS radical was determined according to Samarth et al. [22]. A volume of 88 µL of potassium persulfate (140 mmol/L) was added to 5 mL of ABTS (7 mmol/L). The mixture was stored in an amber bottle in the dark and at room temperature for 16 h. The ABTS radical solution absorbance was adjusted at 0.70 ± 0.05 at the 734 nm in a spectrophotometer. Then, 0.3 mL of film extract solution was added to 3.7 mL of de ABTS radical for 10 min in the dark. After this, the absorbance was measured at 734 nm. The results were expressed in percentage of radical scavenging.

### Antibacterial evaluation

The antibacterial activity of the samples was performed with the shake flask method and disk diffusion method. In addition, Samples were neutralised neutralised using 1.25 mol/L NaOH (5 g NaOH/100 mL H_2_O) for 2 h. For the shake flask method, the bacteria were first incubated in Luria–Bertani medium (LB medium, 1% peptone, 0.5% meat extract, and 1% NaCl, pH 7). The inoculation was then conducted at 37 °C for 24 h under shaking and the obtained bacterial suspension was diluted with the previous peptone medium solution. Afterwards, 0.1 mL of diluted bacteria suspension was cultured in 10 mL liquid peptone medium, and 50 mg of sample was added to the bacterial suspension. The inoculated medium was incubated at 37 °C for 24 h under shaking. After incubation, the antibacterial activity was monitored by measuring the optical density O.D. of the culture medium at 620 nm and calculating the inhibition percentage using the following equation:

1 $$\frac{{O.D.bacteria-O.D.sample}}{{O.D.\,bacteria}} \times 100$$

For the disk diffusion, samples were cut into 6 mm discs which were then sterilised by UV light. Mueller–Hinton agar plates were inoculated with 100 µL of either *E. coli* ATCC 25922 or *S. aureus* NCTC 12981 at a density of 1 × 10^8^ CFU/mL which was dispersed over the entire agar surface with a sterile glass L-shaped spreader. Triplicate discs were aseptically placed on each plate before incubation at 37 °C overnight. After incubation, the diameter of each zone of inhibition observed was measured and recorded.

### Cytotoxic evaluation

Neutralised samples les were sterilised using ethanol for 30 sec, phosphate-buffered saline (PBS) for 30 sec and followed by DMEM media for 30 sec prior to cytotoxic evaluation.

#### Elution assay (cytotoxicity testing)

100 µl of HaCaT cells (1.5 × 10^5^ cells/ml) was seeded in a 96-well plate and incubated overnight at 37 °C. Two different concentrations of extracts, 5 mg/ml and 25 mg/ml were prepared for each sample using DMEM media. They were incubated for 24 h at 37 °C prior to testing. On the following day, the media was removed from the plate and 100 µl of the extract was added into each well and incubated overnight at 37 °C. Following incubation, MTT assay was carried out by treating the cells with 100 µl 0.5 mg/ml MTT solution and incubated for 3.5 h in the 37 °C incubator. The MTT solution was then removed and 100 µl DMSO solution was added into each well. The plate was read at 540 nm using Synergy^TM^ HT BioTek Plate Reader. The cell viability was calculated using following equation:

2 $$\frac{{Abs@540nm\,treated\,cell}}{{Abs@540nm\,untreated\,cell}} \times 100$$

#### Determination of p65-NF-κB protein

5 × 10^5^ cells/well of HaCaT cell line was seeded on the 96-well plate. After overnight incubation at 37 °C, the media was removed and 100 µL of each sample extract at a concentration of 25 mg/ml was added into the well separately and incubated for another 24 h. The amount of total NF-kB p65 and of phosporylated NF-kB p65 proteins presented in the cell lysate was then quantified using an Invitrogen™ NF-kB p65 (Total/Phospho) Human InstantOne ELISA kit, following the manufacturer’s instruction.

## Results and discussion

### Macro- and microscopic analysis

**Fig. 1:**
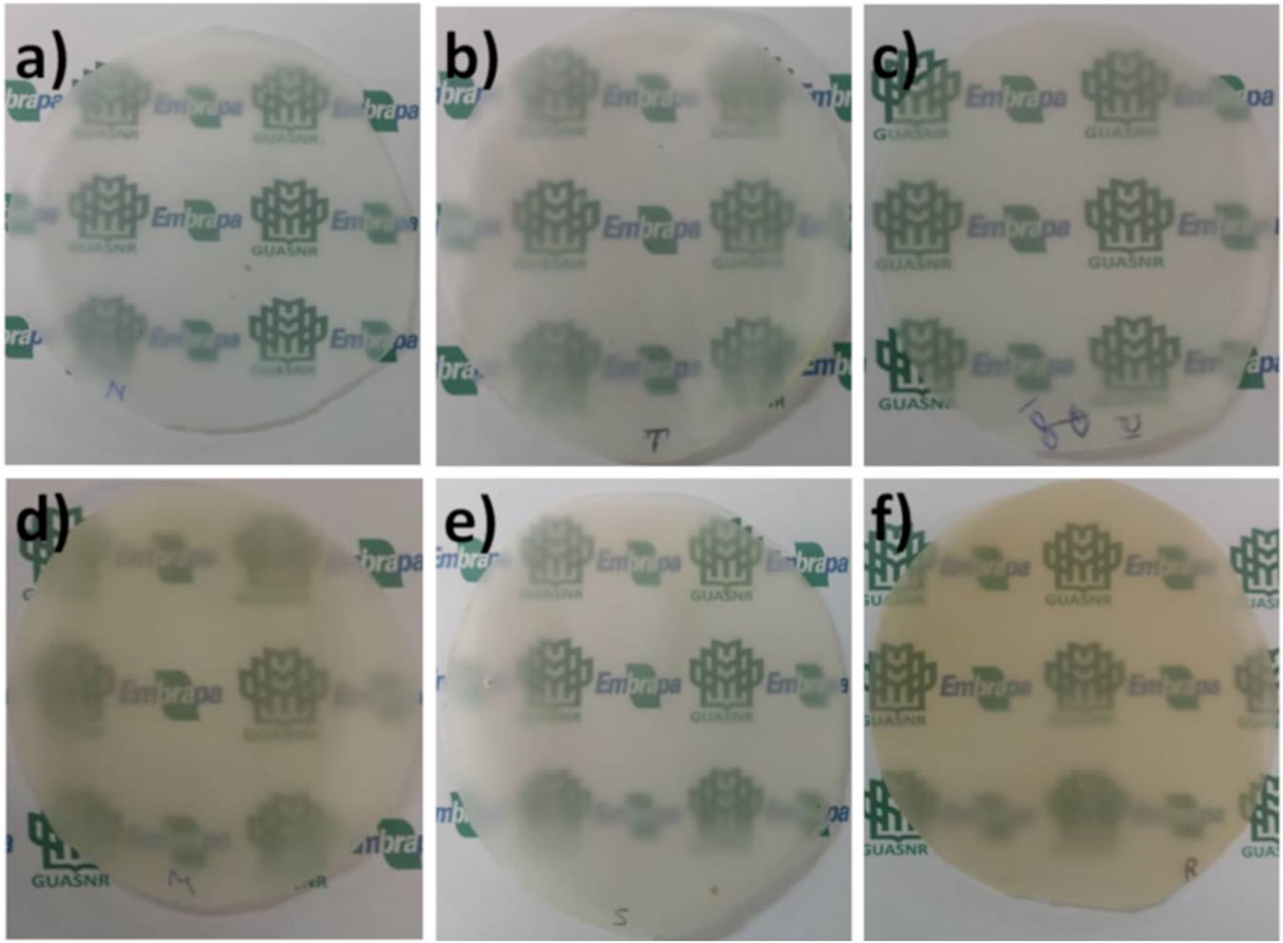
Macroscopic images of samples **a** MFC + GLY, **b** MFC + TA, **c** MFC + TA + GLY, **d** MFC + CHI, **e** MFC + CHI + TA, **f** MFC + TA + CHI + GLY

The macroscopic images of the samples indicate (Fig. 1) that the addition of tannic acid had no difference in the visual opacity; also, chitosan exhibited a yellow vibrance characteristic of this compound, and tannic acid slowly decreased this ochre aspect. The addition of glycerine, even though presented the same opacity as samples without tannic acid, were much more plastic, tested by hand touch; as expected from this material, since glycerine is commonly known to be a good plasticizer. Nonetheless, these relate that the mixture of microfibrillated cellulose with tannic acid led to a more transparent material, which is good for applications such as food packaging or even wound healing. In addition, Chitosan and tannic acid even though is reported to present a yellow colour aspect, the addition of MFC led to a more transparent colour, indicating a strong interaction between these compounds [23].

**Fig. 2:**
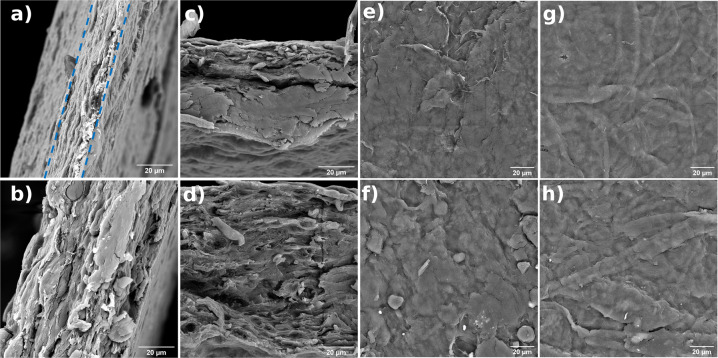
Scanning electron microscope images for cross section (**a**–**d**) and surface (**e**–**h**) for (**a**, **e**) MFC + TA; (**b**, **f**) MFC + TA + GLY; (**c**, **g**) MFC + CHI + TA; (**d**, **h**) MFC + CHI + TA + GLY

All samples presented their internal structure as a lamellar morphology (Fig. 2.a–d), consisting of cellulose stacks, which is a typical structure of nanofibrillated cellulose materials performed by solvent-casting. Samples containing only MFC + TA were very homogeneous, containing well-compacted layers, and the addition of glycerine slightly altered this structure by presenting some rounded objects within the material which could be due to the interaction of tannic acid with glycerine. Nonetheless, the layered structure appears to be more open and not so well distributed, which can be attributed to the glycerine relaxation chain effect. The addition of chitosan still contained a layered ordered spacing but led to a more irregular structure, similar to other previous works [24, 25]; and this ordered structure between these materials relates to a strong interaction [26]. Addition of glycerine led to a more irregular structure, but the size of the layers remained within the same range. The highly viscous MFC material was difficult to cast evenly onto the petri dish, resulting in nonuniform film thicknesses and may explain the morphology from MFC + TA.

The surface of these materials exhibits a “bundled up” surface (Fig. 2.e–h), that is related to the drying method and the spread of the gel when it was poured on petri dish for solvent-casting film formation. There seems to appear some contamination when glycerine was added, which EDX reports to be chlorine and potassium elements. Nonetheless, the structure presents no porosity and is a typical characteristic of films forming by MFC gel solution; but, if aimed for wound healing applications, simple procedures for producing holes using simple apparatus is able to perform a good porosity for air permeation and wound healing [11].

### Microstructure

**Fig. 3:**
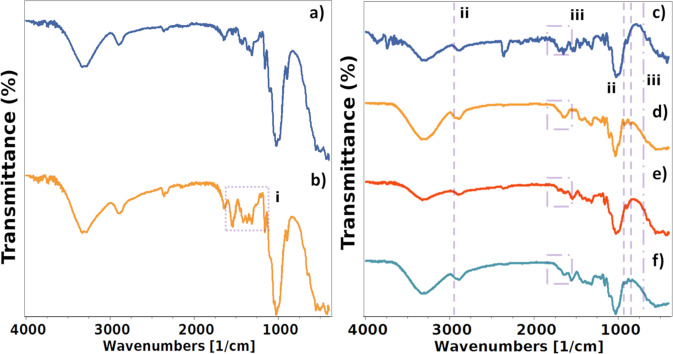
Microfibrillated cellulose films FTIR spectra of **a** MFC, **b** MFC + CHI, **c** MFC + TA, **d** MFC + TA + GLY, **e** MFC + TA + CHI and **f** MFC + TA + CHI + GLY. Region **i** (as round-dot square) denotes the main change when chitosan is added; while **ii** (as dashed lines) when glycerine is added and **iii** (as round-dot square and lines) when TA is added

In order to understand the effect of mixing tannic acid on the structure of the nanocellulose, FTIR was analysed. The majority of microfibrillated cellulose important bands (Fig. 3.a) presented are the region 3700–2980 cm^−1^, corresponding to stretching O–H of intermolecular and intramolecular hydrogen bonds; from 2980–2800 cm^−1^ stretching C–H from alkyl groups; 1630–1650 cm^−1^ assigned to absorption of water; 1455–1314 cm^−1^ bending and scissoring of C–H; 1200–1105 cm^−1^ C–O–C symmetric and asymmetric groups [27]. Finally, 1026 and 896 cm^−1^ corresponding to -CO and -CH groups in cellulose, respectively [28]. When chitosan was incorporated into MFC films, the spectra remained similar with few shifts occurring (OH region and carboxylic groups) but the addition of new peaks was detected (Fig. 3.b–i); such as the region of 1593–1501 cm^−1^, characteristic absorption bands of chitosan in 1556 and 1541 cm^−1^ which are assigned to the stretching vibrations of amino groups. In addition to another characteristic chitosan band assigned to CH_2_ scissoring at 1418 cm^−1^ [29].

When tannic acid was incorporated into the films, a large region is modified by the presence of its groups (Fig. 3.c–f–iii) from 1737–1671 cm^−1^, corresponding to the vibration of C=O phenolic esters, and a small band that appeared around the region of 770–750 cm^−1^ (Fig. 3.c–f–iii) corresponding to symmetric skeletal vibration [30]. Besides, the most characteristics bands are within the region of the MFC and chitosan which is difficult to determine; though the strongest band characteristic of tannic acid around 1043–1030 cm^−1^, corresponding to in plane C–H deformation – shifted to the tannic acid band indicating as well the incorporation of tannic acid in the structure [31]. The interaction of tannic acid–chitosan is mainly due to a complex electrostatic behaviour; they could develop extensive hydrogen bonds acting as crosslinker for chitosan [32].

When glycerine was added to the structure of these films, besides increasing the OH bands due to the increase ability of glycerine to absorb water, they also showed new bands characteristic of glycerine. These bands are 2935 cm^−1^ assigned to an aliphatic group, 924 cm^−1^ bound –OH in glycerine, and 849 cm^−1^ stretching vibrations of C–O–C groups [33].

Therefore, besides confirming the incorporation of all added products by the identified characteristic bands of each compound, the FTIR identified that tannic acid can also enhance the structure of chitosan—more detailed bands, which has been also shown by other authors [6, 7]. Furthermore, tannic acid can weakly attenuate the heavily plasticising effect of glycerine to produce mouldable films with desirable mechanical properties.

### Thermal characterisation

**Fig. 4:**
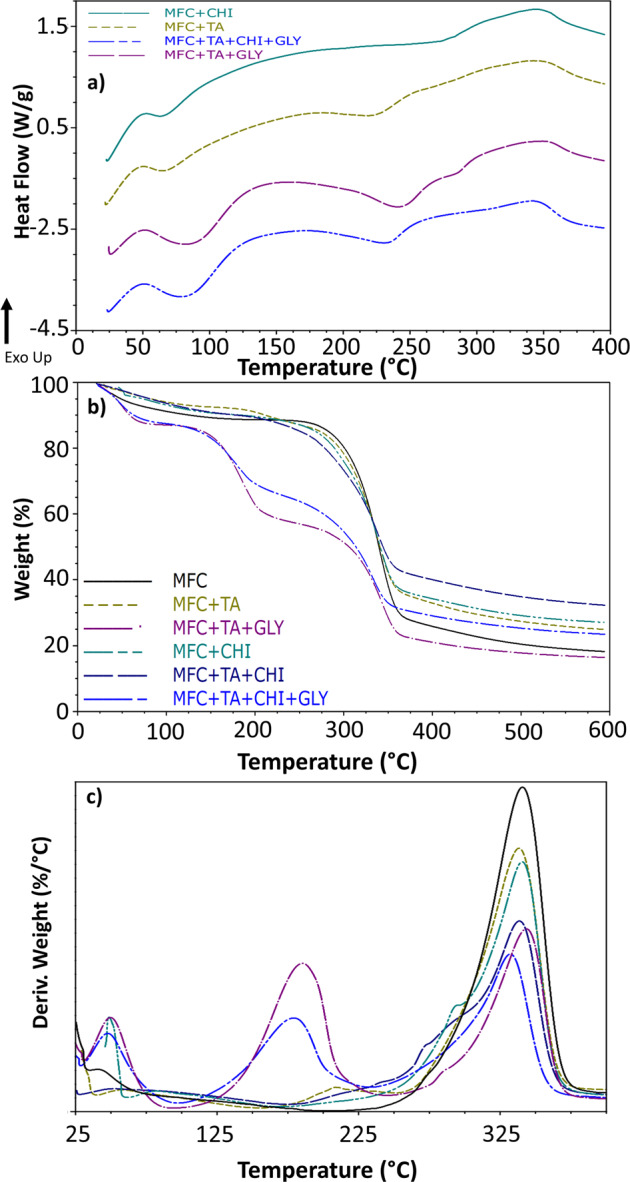
**a** Differential scanning calorimetry and **b** thermogravimetric analysis with its **c** first derivative for the microfibrillated cellulose films

In order to evaluate the thermal properties of these films, differential scanning calorimetry was performed (Fig. 4.a). The thermo profile shows that the addition of chitosan to the MFC film results in one endothermic peak between 50–125 °C due to the evaporation of water due to physical changes during N-deacetylation and carboxymethylation [34]. The second event is a large exothermic peak related to the degradation of chitosan material 230–350 °C [35], finally within 300–400 °C is the degradation of MFC components—cellulose degradation of glycosyl units and oxidation breakdown, forming low molecular weight products [20].

The addition of tannic acid, besides also exhibiting the endothermic peak of water evaporation, presented a peak around 200–270 °C which is related to degradation of tannins and partial breakdown of intermolecular bonding [36]. In addition, modified tannins with longer chain ester can exhibit melting features [37], which occurs from the bonding interaction of tannic acid with cellulose and/or chitosan.

Finally, when glycerine is added to the structure it reduces the intermolecular forces, while also increases the mobility of polymer chains [38]. The glycerol promoted the hygroscopicity of the cellulose films due to its hydrophilic character [39] and resulted in a strong impact on the thermal stability of these films.

The thermogravimetric analysis (Fig. 4.b) of these films exhibits that no significant variation occurs with the thermal stability of these films without the addition of glycerine, while its addition further decreases their stability, as observed with the DSC results. Nonetheless, there are three main weight loss steps observed (Fig. 3.c), the first from 25–100 °C related to the water absorbed and other events depicted on DSC; also, a weight loss at 125–240 °C attributed to glycerol, and the biggest within 250–375 °C attributed to cellulose and the other compounds degradation. Besides these, small events can be seen at 175–250 °C for samples containing tannic acid, which further proves the melting features due to bonding. In addition to other related small events within 225–275 °C, attributed to chitosan degradation bonded within cellulose.

### Mechanical properties and antioxidant analysis

**Fig. 5:**
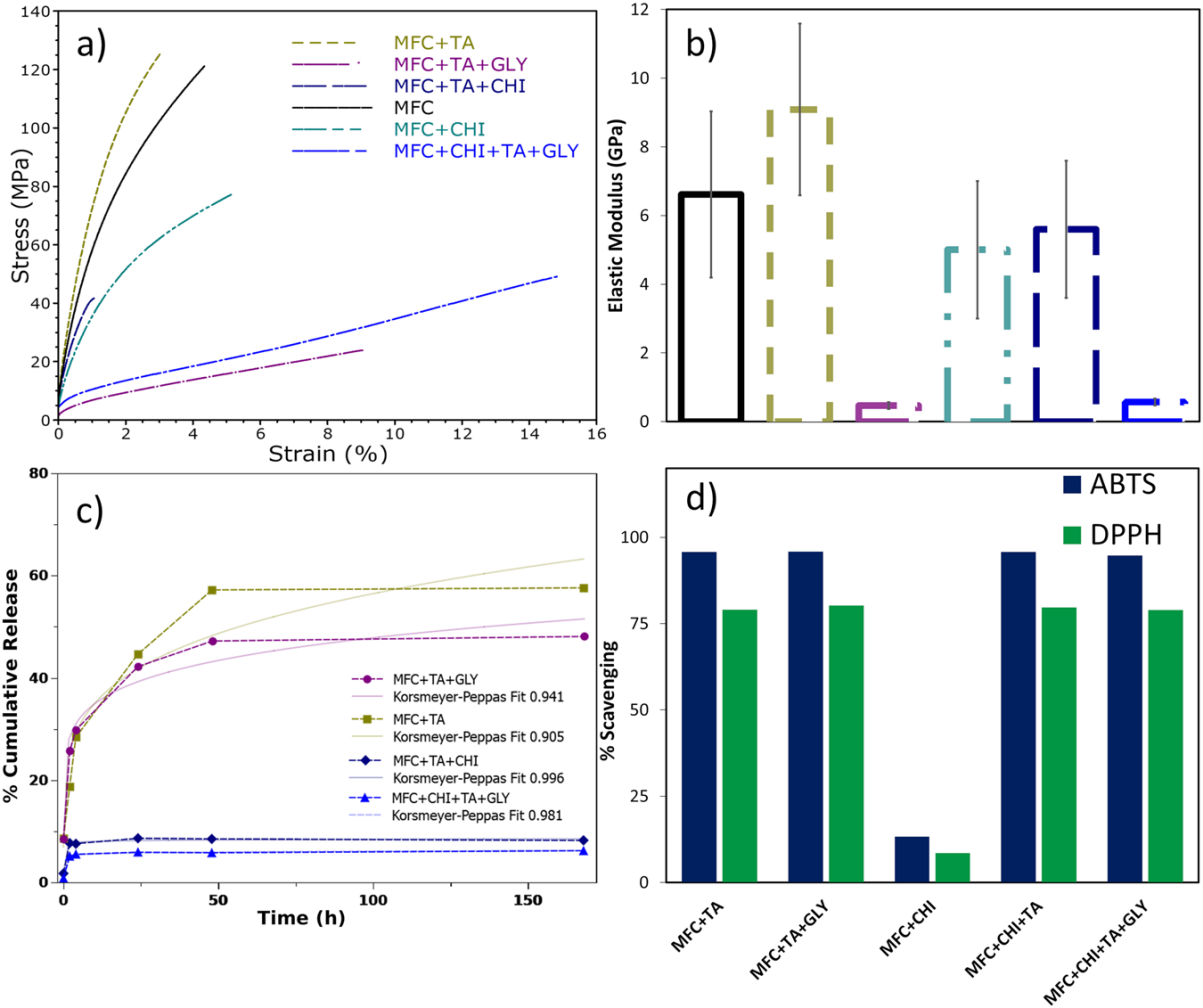
**a** Stress–strain curves for the MFC films performed by DMA and its **b** elastic modulus; **c** tannic acid % cumulative release profile and **d** ABTS and DPPH antioxidant scavenging activity

It is already known that films produced with microfibrillated cellulose are very strong from previous tensile tests [11, 20] (Fig. 5.a), while also able to increase this property when incorporated into many materials, such as chitosan films [40], at the expense of becoming less flexible and more brittle. Nonetheless, when these materials were incorporated with tannic acid, a more brittle material resulted. This can be attributed to its crosslinking nature, where multiple hydrogen bonds occurred within tannic acid and carboxyl groups from the cellulose or chitosan [41].

Values of MFC films elastic modulus (Fig. 5.b) for bleached kraft cellulose from *Eucalyptus sp*. can be up to 5 GPa. The films reported herein also presented similar values, and although the addition of chitosan and tannic acid presented lower and higher mean values respectively, these were not statistically significant. Finally, when glycerine is added, the plasticizer effect had a huge impact on the tensile tests, reducing the tensile strength and increasing the elongation at break. Exhibiting an elastic modulus of 0.5 GPa, a phenomenon already known due to its plasticisation, and already reported before [39].

Tannic acid release rate depends on the structure where it is synthesised; and, in such cases, when incorporated with microfibrillated cellulose (Fig. 5.c) a sustained release is possible to achieve for up to a week, and the addition of glycerine slightly decrease the release rate. However, ~30% of tannic acid as burst release occurs within the first 4 h achieving a new release profile thereafter, this could be related to the interaction of tannic acid with cellulose groups as described by the FTIR, whereas relaxation of macromolecular chains may be involved and presented a somewhat low fit for Korsmeyer–Peppas release.

Nonetheless, when incorporated with chitosan, a heavily decrease occurs to their release profile, characteristic of a potential crosslink and already described by literature. In such cases, the tannic acid is well linked with the cellulose and chitosan groups so that there are few nonbounded chemical groups that affect their release profile. In addition, the release model is more accurately described as a “Less Fickian” model, characterised by release coefficients outside the limits of the model (*n* < 0.5 where *n* was <0.3 in our work), and the drug release is still considered as a Fickian diffusion. Chitosan films have been reported to modify the structure in order to show such increased fast release [42], also, the kinetic swelling parameter of such chitosan films by solvent-casting dissolved from acetic acid also exhibit this character [43]. In addition to hydrogels [44], and previously using MFC [45], such models are important in order to study the effective drug delivery in vivo, so that smart polymers can achieve an ideal release profile by electrically controlling the rate based on such models [46], or by pH [47].

Even though the addition of tannic acid led to a more brittle film and a strong interaction with the other components, it did not decrease its antioxidant nature when incorporated into the films (Fig. 5.d). The important aspect of the antioxidant nature of tannic acid is the ability to scavenge free radicals in foods or biological systems, which are associated with many degenerative conditions [2, 4]. The test then evaluates its antioxidant power by measuring the decrease in the absorbance of DPPH at 515 nm – specifically, the tannins presented in the film was measured with a solution of 0.1 mL that contained 25 mg of film which was extracted in a 3 ml of solvent and was able to reduce 80% from a solution containing 3.9 mL that contained 24 mg/L of DPPH. The concentration of tannic acid added in the films, 10 %wt, has also been reported by other authors to be effective due to the effective scavenging ability of its polyphenol [48].

In the case of ABTS, cation radicals scavenging activity, the test also evaluates the antioxidant power after reaction of ABTS with tannic acid by measuring its decrease at 734 nm, and it presents scavenging values of 90%. Therefore, the tannic acid in the films could still remain active as antioxidant in order to scavenge free radicals, and is reported to have positive effects on wound healing processes [4, 49]. Higher values of ABTS has also been reported by other authors with the usage of tannic acid incorporated into nanocellulose [50]. The antioxidant effect of tannic acid in vivo has been reported to reduce the lipid peroxidation, COX-2, inducible nitric oxide synthase (i-NOS), and PCNA expression [51] and these effects are known to be the most important for its anticancer property.

### Antibacterial activity

**Fig. 6:**
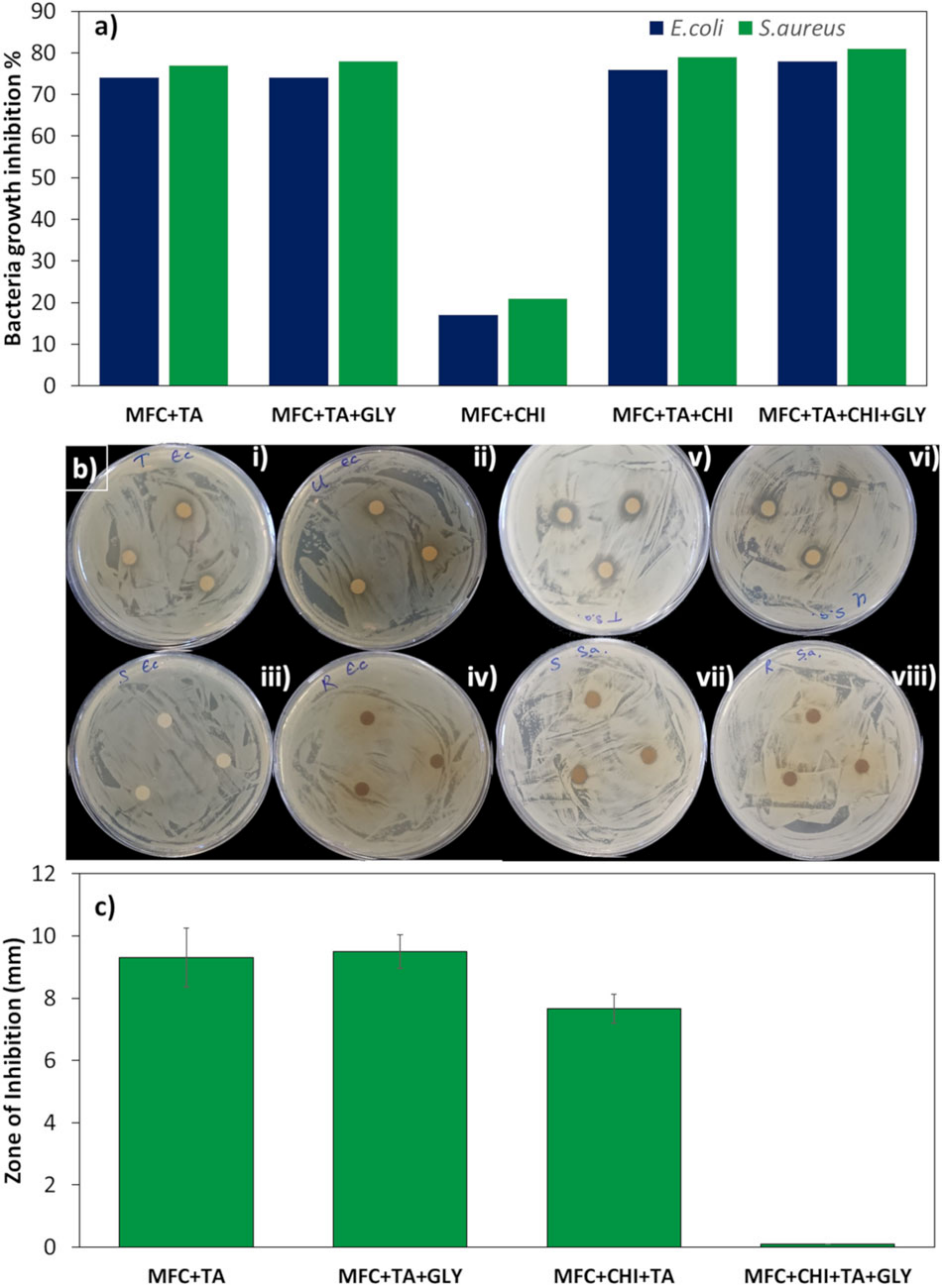
**a** Bacteria growth inhibition of 25 mg samples containing tannic acid; **b** disk diffusion test macroscopic images for bacteria inhibition after 24 h for *E. coli* (**i**–**iv**) and *S. aureus* (**v**–**viii**) where (**i**, **v**) MFC + TA, (**ii**, **vi**) MFC + TA + GLY, (**iii**, **vii**) MFC + CHI + TA, (**iv**–**viii**) MFC + TA + CHI + GLY and **c** zone of inhibition diameter for *S. aureus*

In order to understand if tannic acid can still present antibacterial activity when incorporated into the films, tests of disk diffusion and shake flask were performed in *E. coli* and *S. aureus* (Fig. 6). For the shake flask test (Fig. 6.a), all samples containing tannic acid presented antibacterial activity over 70% for both bacteria; while *S. aureus* had 10% more effectiveness, and at this concentration, is within values found by another work using tannic acid [52, 53]. In the case of disk diffusion (Fig. 5.b–c), the results presented no inhibition of growth when using *E. coli*; however, for the samples MFC + TA and MFC + TA + GLY, thinning of the bacterial growth was observed and may indicate a possible inhibitory effect if the concentration of the antimicrobial agent or its ability to elute was increased. In regard to *S. aureus*, bacterial growth inhibition was observed for the majority of the samples, and overall, the values of disk diffusion bacteria inhibition for tannic acid was similar to other reports using the same concentration [53, 54].

Since these samples were neutralised before testing for antibacterial, the presented low values may well be related to incorporated tannic acid, compared to acetic acid which is reported to have higher antibacterial effect for these bacteria even at lower concentrations [55, 56]. Even though chitosan has been reported to have antibacterial effect [57], the values found in this work for samples containing cellulose with chitosan had a weak antibacterial activity. When they were incorporated with tannic acid, the antibacterial effect is still lower than when compared to samples without chitosan. We attributed this possibility to the crosslinking mechanism that occurred within cellulose and chitosan, leading to fewer groups being able to inhibit bacteria due to the structure. In addition, these same groups could have interacted with tannic acid, also decreasing their potential inhibition.

The higher activity against *S. aureus* is due to the mechanism of polyphenols that directly binds on peptidoglycan layer of Gram-positive bacteria. In the case of Gram-negative species, this layer is thinner and the components of cell wall cannot bind to polyphenols, leading to lower values against *E. coli* [58]. Although the antibacterial effect of many nanomaterials generally involves generation of reactive oxygen species to induce oxidative stress to the bacteria cell wall [59], tannic acid presents a unique behaviour. This is related to be due to chelation of iron from the medium, which leads it to be unavailable for the bacteria growth [60]. Therefore, due to many antibacterial mechanisms that tannic acid possess, it is a bactericide for a broad spectrum of bacteria [2].

### Cell viability assays

**Fig. 7:**
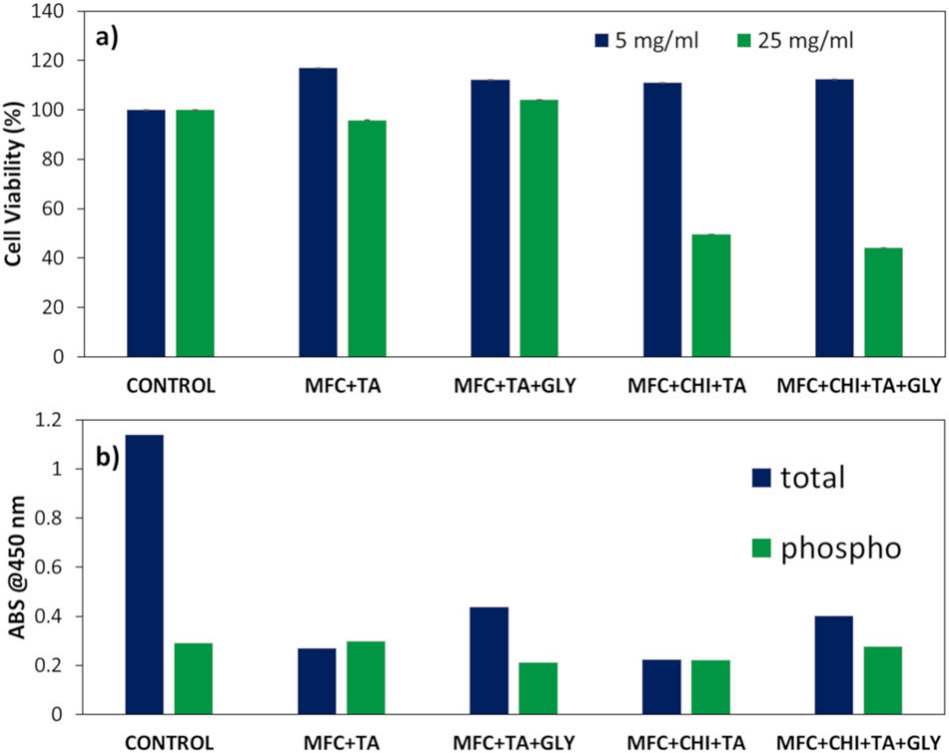
**a** Cell viability elution assay for two concentrations of film density and **b** phosphorylated NF-κB activity for the studied samples containing tannic acid at 25 mg/ml

In order to investigate if the tannic acid could still promote cell growth as a wound healing material, they were submitted to cell viability essays (Fig. 7.a) using HaCaT cells - epidermal keratinocytes. The cell metabolic activity (elution essay) results exhibit that samples containing tannic acid presented higher viability for HaCaT cells compared to control, and are all larger than 110%, considered as non-toxic at the concentration of 5 mg/ml. The crosslink between chitosan and tannic acid could decrease the viability seen by these results, lowering the exposure of some characteristic groups to cell interaction. Besides, the addition of glycerine did not alter its high metabolic activity to cells.

However, increasing the concentration of tannic acid, a heavily increase in toxicity occurs, whereas samples of MFC + CHI + TA and MFC + CHI + TA + GLY had a severe effect on cell proliferation. Instead, samples MFC + TA and MFC + TA + GLY can still be considered as non-toxic at a higher concentration of 25 mg/ml, this could again be attributed to the interaction structure, and how well tannic acid binds to the other components. Additionally, the purification method used to eliminate the acidic condition may have been ineffective when chitosan was added to the structure. However, increase in dosages of tannic acid has been also shown to be toxic as reported by other authors [61, 62].

This effect can be further seen by the test with Nuclear factor kappa B (NF-κB) p65 signalling (Fig. 7.b). NF-κB is a transcription factor related to inflammatory, proliferation and apoptosis processes and is strongly implicated in initiation of pro-inflammatory target gene expression, e.g., IL-6, IL-8 or COX-2 [63], it also plays a crucial role in fibroblast healing and regeneration for various biomaterials [64]. In the case of tannic acid, it is reported that it can block NF-kB activation in vitro causing a decline in the production of inflammatory mediators [65]; thereby inhibiting the expression of adhesion molecules. Therefore, samples containing tannic acid had a reduction on the expression of this factor, and there seems to be an increase when glycerine is added, which may be related to a slower release of free tannic acid compound in the samples.

The healing mode of action from tannic acid involves elimination of water into cells—due to its astringent action which contracts the fibres by decreasing haemorrhage and facilitating healing [49]. Nonetheless, it is important to state that the method of application in vivo for tannic acid – subcutaneously injected or topically - produces different results, if subcutaneously injected it can cause a severe toxicity [66] whereas in subcutaneous administration it possesses agreeable outcomes [67].

## Conclusions

Microfibrillated cellulose containing tannic acid presents a well-formed internal layered structure based on electrostactic interaction, confirmed by IR from new bands formed such as C=O phenolic ester groups, while also presenting an increased tensile resistance characteristic of a more rigid structure (up to 8 GPa). Chitosan incorporation presented characteristic bands, such as amino groups, from the IR spectra and led to a small reduction in the mechanical properties (around 4–6 GPa). Thermal degradation had no significant variation within the films that did not contained glycerine, but characteristic degradation steps of chitosan and tannic acid were observed in DTA. The addition of glycerine further decreases the thermal and mechanical properties by reducing the intermolecular forces and increase mobility chains.

The addition of tannic acid promoted good antioxidant scavenging activity values, achieving ~90% for ABTS and ~75% for DPPH. In addition, the highest drug release was achieved solely with MFC + TA (~60%) and the addition of chitosan led to a heavy decrease (~8%) attributed to the crosslink that occurred within the polymeric materials. Finally, the release rate and amount of tannic acid proved to be effective at inhibiting common pathogens though only as shake flask methodology, while disk diffusion only presented inhibition for *S. aureus*. Lastly, tannic acid presented to be viable for specific samples at higher concentration – MFC + TA and MFC + TA + GLY – but with an inhibition of the phosphorylated NF-κB activity, characteristic of tannic acid compound. Nonetheless, it appears to slightly decrease this inhibition when blended with glycerine. Therefore, the release rate of this material needs to be a point of concern because it can also harm the cells, so materials that can entrap this compound to a slower release can be beneficial when considering its addition on polymeric systems such as glycerine.

## Data Availability

Data will be made available upon request.

## References

[1] Graham N, Gang F, Fowler G, Watts M (2008). Characterisation and coagulation performance of a tannin-based cationic polymer: a preliminary assessment. Colloids Surf A Physicochem Eng Asp.

[2] Kim TJ, Silva JL, Kim MK, Jung YS (2010). Enhanced antioxidant capacity and antimicrobial activity of tannic acid by thermal processing. Food Chem.

[3] Pulido R, Bravo L, Saura-Calixto F (2000). Antioxidant activity of dietary polyphenols as determined by a modified ferric reducing/antioxidant power assay. J Agric Food Chem.

[4] Antonia de SL, Laynne H, de CL, Davi da S, Livio CCN, Jose ADL (2015). Incorporation of tannic acid in formulations for topical use in wound healing: a technological prospecting. Afr J Pharm Pharm.

[5] Liu H-F, Zhang F, Lineaweaver WC (2017). History and advancement of burn treatments. Ann Plast Surg.

[6] Rivero S, García MA, Pinotti A (2010). Crosslinking capacity of tannic acid in plasticized chitosan films. Carbohydr Polym.

[7] Rubentheren V, Ward TA, Chee CY, Nair P (2015). Physical and chemical reinforcement of chitosan film using nanocrystalline cellulose and tannic acid. Cellulose.

[8] Rubentheren V, Ward TA, Chee CY, Nair P, Salami E, Fearday C (2016). Effects of heat treatment on chitosan nanocomposite film reinforced with nanocrystalline cellulose and tannic acid. Carbohydr Polym.

[9] Abdelraof M, Hasanin MS, Farag MM, Ahmed HY (2019). Green synthesis of bacterial cellulose/bioactive glass nanocomposites: effect of glass nanoparticles on cellulose yield, biocompatibility and antimicrobial activity. Int J Biol Macromol.

[10] Yang R-T, Yu H-Y, Song M-L, Zhou Y-W, Yao J-M (2016). Flower-like zinc oxide nanorod clusters grown on spherical cellulose nanocrystals via simple chemical precipitation method. Cellulose.

[11] Claro FC, Jordão C, de Viveiros BM, Isaka LJE, Villanova JA, Magalhães WLE (2020). Low cost membrane of wood nanocellulose obtained by mechanical defibrillation for potential applications as wound dressing. Cellulose.

[12] Taheri P, Jahanmardi R, Koosha M, Abdi S (2020). Physical, mechanical and wound healing properties of chitosan/gelatin blend films containing tannic acid and/or bacterial nanocellulose. Int J Biol Macromol.

[13] Missio AL, Mattos BD, Ferreira D, de F, Magalhães WLE, Bertuol DA (2018). Nanocellulose-tannin films: From trees to sustainable active packaging. J Clean Prod.

[14] Kumar S, Mukherjee A, Dutta J (2020). Chitosan based nanocomposite films and coatings: emerging antimicrobial food packaging alternatives. Trends Food Sci Technol.

[15] Ojeda-Hernández DD, Canales-Aguirre AA, Matias-Guiu J, Gomez-Pinedo U, Mateos-Díaz JC (2020). Potential of chitosan and its derivatives for biomedical applications in the central nervous system. Front Bioeng Biotechnol.

[16] Moeini A, Pedram P, Makvandi P, Malinconico M, Gomez d’Ayala G (2020). Wound healing and antimicrobial effect of active secondary metabolites in chitosan-based wound dressings: a review. Carbohydr Polym.

[17] Lamarra J, Rivero S, Pinotti A (2020). Nanocomposite bilayers based on poly(vinyl alcohol) and chitosan functionalized with gallic acid. Int J Biol Macromol.

[18] Mujtaba M, Morsi RE, Kerch G, Elsabee MZ, Kaya M, Labidi J (2019). Current advancements in chitosan-based film production for food technology; a review. Int J Biol Macromol.

[19] Dharupaneedi SP, Anjanapura RV, Han JM, Aminabhavi TM (2014). Functionalized graphene sheets embedded in chitosan nanocomposite membranes for ethanol and isopropanol dehydration via pervaporation. Ind Eng Chem Res.

[20] de Lima GG, Ferreira BD, Matos M, Pereira BL, Nugent MJD, Hansel FA (2020). Effect of cellulose size-concentration on the structure of polyvinyl alcohol hydrogels. Carbohydr Polym.

[21] Siripatrawan U, Harte BR (2010). Physical properties and antioxidant activity of an active film from chitosan incorporated with green tea extract. Food Hydrocoll.

[22] Samarth RM, Panwar M, Kumar M, Soni A, Kumar M, Kumar A (2008). Evaluation of antioxidant and radical-scavenging activities of certain radioprotective plant extracts. Food Chem.

[23] Roy S, Zhai L, Kim HC, Pham DH, Alrobei H, Kim J (2021). Tannic-acid-cross-linked and TiO2-nanoparticle-reinforced chitosan-based nanocomposite film. Polymers.

[24] Hänninen A, Sarlin E, Lyyra I, Salpavaara T, Kellomäki M, Tuukkanen S (2018). Nanocellulose and chitosan based films as low cost, green piezoelectric materials. Carbohydr Polym.

[25] Franco TS, Amezcua RMJ, Rodrìguez AV, Enriquez SG, Urquíza MR, Mijares EM (2020). Carboxymethyl and Nanofibrillated Cellulose as Additives on the Preparation of Chitosan Biocomposites: Their Influence Over Films Characteristics. J Polym Environ.

[26] Sundaram J, Pant J, Goudie MJ, Mani S, Handa H (2016). Antimicrobial and physicochemical characterization of biodegradable, nitric oxide-releasing nanocellulose–chitosan packaging membranes. J Agric Food Chem.

[27] Summerscales J, Gwinnett C. Forensic identification of bast fibres. In Biocomposites High-Performance Applications. Elsevier; 2017. p. 125–64.

[28] Hospodarova V, Singovszka E, Stevulova N (2018). Characterization of cellulosic fibers by FTIR spectroscopy for their further implementation to building materials. Am J Anal Chem.

[29] Hassan MM, Mohamed MH, Udoetok IA, Steiger BGK, Wilson LD (2020). Sequestration of sulfate anions from groundwater by biopolymer-metal composite materials. Polymers.

[30] Falcão L, Araújo MEM (2014). Application of ATR–FTIR spectroscopy to the analysis of tannins in historic leathers: the case study of the upholstery from the 19th century Portuguese Royal Train. Vib Spectrosc.

[31] Falcão L, Araújo M (2018). Vegetable Tannins used in the manufacture of historic leathers. Molecules.

[32] Rivero S, García MA, Pinotti A (2012). Heat treatment to modify the structural and physical properties of chitosan-based films. J Agric Food Chem.

[33] Kachel-Jakubowska M, Matwijczuk A, Gagoś M (2017). Analysis of the physicochemical properties of post-manufacturing waste derived from production of methyl esters from rapeseed oil. Int Agrophysics.

[34] Fai AEC, Stamford TCM, Stamford-Arnaud TM, Santa-Cruz PD, Silva MCF, da, Campos-Takaki GM (2011). Physico-chemical characteristics and functional properties of chitin and chitosan produced by Mucor circinelloides using yam bean as substrate. Molecules.

[35] Guinesi LS, Cavalheiro ÉTG (2006). The use of DSC curves to determine the acetylation degree of chitin/chitosan samples. Thermochim Acta.

[36] Pantoja-Castro MA, González-Rodríguez H (2011). Study by infrared spectroscopy and thermogravimetric analysis of Tannins and Tannic acid. Rev Latinoam Qu¡mica.

[37] Grigsby W, Bridson J, Lomas C, Elliot J-A (2013). Esterification of condensed tannins and their impact on the properties of poly(lactic acid). Polymers.

[38] Arık Kibar EA, Us FThermal (2013). mechanical and water adsorption properties of corn starch–carboxymethylcellulose/methylcellulose biodegradable films. J Food Eng.

[39] Ma X, Qiao C, Wang X, Yao J, Xu J (2019). Structural characterization and properties of polyols plasticized chitosan films. Int J Biol Macromol.

[40] Fernandes SCM, Freire CSR, Silvestre AJD, Pascoal Neto C, Gandini A, Berglund LA (2010). Transparent chitosan films reinforced with a high content of nanofibrillated cellulose. Carbohydr Polym.

[41] Hager A-S, Vallons KJR, Arendt EK (2012). Influence of gallic acid and tannic acid on the mechanical and barrier properties of wheat gluten films. J Agric Food Chem.

[42] Paiva Filho JC, de Morais SM, Nogueira Sobrinho AC, Cavalcante GS, da Silva NA, da S. Abreu FOM (2019). Design of chitosan-alginate core-shell nanoparticules loaded with anacardic acid and cardol for drug delivery. Polímeros.

[43] Gierszewska-Druzynska M, Ostrowska-Czubenko J (2012). Mechanism of water diffusion into noncrosslinked and ionically crosslinked chitosan membranes. Prog Chem Appl Chitin its Deriv.

[44] Ilić-Stojanović S, Nikolić L, Nikolić V, Petrović S, Oro V, Mitić Ž (2021). Semi-Crystalline copolymer hydrogels as smart drug carriers: in vitro thermo-responsive naproxen release study. Pharmaceutics.

[45] Aliabadi M, Chee BS, Matos M, Cortese YJ, Nugent MJD, de Lima TAM (2020). Yerba mate extract in microfibrillated cellulose and corn starch films as a potential wound healing bandage. Polymers.

[46] Patil SB, Inamdar SZ, Das KK, Akamanchi KG, Patil AV, Inamadar AC (2020). Tailor-made electrically-responsive poly(acrylamide)-graft-pullulan copolymer based transdermal drug delivery systems: synthesis, characterization, in-vitro and ex-vivo evaluation. J Drug Deliv Sci Technol.

[47] de Lima GG, Chee BS, Moritz VF, Cortese YJ, Magalhães WLE, Devine DM (2019). The production of a novel poly(vinyl alcohol) hydrogel cryogenic spheres for immediate release using a droplet system. Biomed Phys Eng Express.

[48] Li P, Sirviö JA, Haapala A, Khakalo A, Liimatainen H (2019). Anti-oxidative and UV-absorbing biohybrid film of cellulose nanofibrils and tannin extract. Food Hydrocoll.

[49] Xu F, Weng B, Gilkerson R, Materon LA, Lozano K (2015). Development of tannic acid/chitosan/pullulan composite nanofibers from aqueous solution for potential applications as wound dressing. Carbohydr Polym.

[50] Ge W, Cao S, Shen F, Wang Y, Ren J, Wang X (2019). Rapid self-healing, stretchable, moldable, antioxidant and antibacterial tannic acid-cellulose nanofibril composite hydrogels. Carbohydr Polym.

[51] Costa A, Bonner MY, Arbiser JL (2016). Use of polyphenolic compounds in dermatologic oncology. Am J Clin Dermatol.

[52] Huang J, Cheng Y, Wu Y, Shi X, Du Y, Deng H (2019). Chitosan/tannic acid bilayers layer-by-layer deposited cellulose nanofibrous mats for antibacterial application. Int J Biol Macromol.

[53] Zhang Z-Y, Sun Y, Zheng Y-D, He W, Yang Y-Y, Xie Y-J (2020). A biocompatible bacterial cellulose/tannic acid composite with antibacterial and anti-biofilm activities for biomedical applications. Mater Sci Eng C.

[54] Higazy A, Hashem M, ElShafei A, Shaker N, Hady MA (2010). Development of anti-microbial jute fabrics via in situ formation of cellulose–tannic acid–metal ion complex. Carbohydr Polym.

[55] Fraise AP, Wilkinson MAC, Bradley CR, Oppenheim B, Moiemen N (2013). The antibacterial activity and stability of acetic acid. J Hosp Infect.

[56] Roe AJ, O’Byrne C, McLaggan D, Booth IR (2002). Inhibition of Escherichia coli growth by acetic acid: a problem with methionine biosynthesis and homocysteine toxicity. Microbiology.

[57] Goy RC, Assis OBG (2014). Antimicrobial analysis of films processed from chitosan and N,N,N-trimethylchitosan. Braz J Chem Eng.

[58] Perelshtein I, Ruderman E, Francesko A, Fernandes MM, Tzanov T, Gedanken A (2014). Tannic acid NPs – Synthesis and immobilization onto a solid surface in a one-step process and their antibacterial and anti-inflammatory properties. Ultrason Sonochem.

[59] Kannan K, Radhika D, Nesaraj A, Kumar Sadasivuni K, Reddy KR, Kasai D (2020). Photocatalytic, antibacterial and electrochemical properties of novel rare earth metal oxides-based nanohybrids. Mater Sci Energy Technol.

[60] Chung K-T, Lu Z, Chou M (1998). Mechanism of inhibition of tannic acid and related compounds on the growth of intestinal bacteria. Food Chem Toxicol.

[61] Silva GA, Vaz CM, Coutinho OP, Cunha AM, Reis RL (2003). In vitro degradation and cytocompatibility evaluation of novel soy and sodium caseinate-based membrane biomaterials. J Mater Sci Mater Med.

[62] Sahiner M, Sagbas S, Bitlisli BO (2015). p(AAm/TA)-based IPN hydrogel films with antimicrobial and antioxidant properties for biomedical applications. J Appl Polym Sci.

[63] Liu T, Zhang L, Joo D, Sun S-C (2017). NF-κB signaling in inflammation. Signal Transduct Target Ther.

[64] Park YR, Sultan MT, Park HJ, Lee JM, Ju HW, Lee OJ (2018). NF-κB signaling is key in the wound healing processes of silk fibroin. Acta Biomater.

[65] Heng MCY, Allen SG, Kim A (1990). Tannic-acid staining material on high endothelial venules and lymphocytes in skin and peripheral lymph nodes in Staphylococcus aureus-associated erythroderma. Clin Exp Dermatol.

[66] Cameron GR, Milton RF, Allen JW (1943). Toxicity of tannic acid. Lancet.

[67] Halkes SBA, van den Berg AJJ, Hoekstra MJ, du Pont JS, Kreis RW (2002). Transaminase and alkaline phosphatase activity in the serum of burn patients treated with highly purified tannic acid. Burns.

